# Outcomes of an interdisciplinary work rehabilitation program

**DOI:** 10.3233/WOR-193012

**Published:** 2019-11-21

**Authors:** Mitchell R. Voss, Jennifer K. Homa, Maharaj Singh, Jennifer A. Seidl, Wesley E. Griffitt

**Affiliations:** a Physical Medicine and Rehabilitation, Aurora BayCare Medical Center, Green Bay, WI, USA; b Aurora Research Institute, Aurora Health Care, Milwaukee, WI, USA; c Physical Medicine and Rehabilitation, Aurora Health Care, Milwaukee, WI, USA; d Neurosurgery, BayCare Clinic, Green Bay, WI, USA

**Keywords:** Return to work, intervention, strength, work rehabilitation

## Abstract

**BACKGROUND:**

Work rehabilitation programs were developed to help workers with an injury return to work (RTW). While studies have examined intervention characteristics, prognostic factors, and disability level, there is little or no research examining interdisciplinary interventions, lifting capacity/strength and the level of a patient’s RTW status (e.g., not working, new job, or ongoing restrictions) at the time of discharge.

**OBJECTIVE:**

To evaluate outcomes (RTW status and lifting capacity/strength changes) of an interdisciplinary work rehabilitation program and examine whether time off work prior to the program and type of injury were related to RTW status and strength changes.

**METHODS:**

A retrospective database analysis was conducted with a sample of 495 participants (M_age_ = 44.44 years, SD = 10.13) of which 375 (76%) were male. Participants were workers with injuries who participated in an interdisciplinary work rehabilitation program from 2006 to 2010.

**RESULTS:**

A significantly higher number of participants were working at the end of the program than at the beginning (83.9% vs. 31.6%, *p* < 0.0001). Mean strength was higher at the time of discharge compared to at admission (*p* < 0.0001). The participants that did not RTW had had significantly more days off work prior to the program (U = 11757, z = –3.152, *p* = 0.002). The type of injury was not related to strength at the time of discharge.

**CONCLUSIONS:**

Findings suggest the interdisciplinary program is associated with positive outcomes and early intervention may be an important factor when treating patients with work-related injuries.

## 1 Introduction

Work rehabilitation refers to a wide variety of programs and interventions designed to help the worker with an injury return to work (RTW) [1]. The three primary work rehabilitation program types include work conditioning, work hardening, and transitional work programs. Work conditioning utilizes an approach promoting work simulation and personalized treatment for 2 to 4 hours per session occurring 3 to 5 days a week [1]. Work hardening includes a multidisciplinary approach and can involve psychological services, job coaching, ergonomic evaluation, and transitional work services [1]. Workers participate in work hardening 2 or more hours a day 5 days a week [1]. Transitional work programs focus on the specific work tasks and work environment, with recommendations by the treatment team for task and environmental modifications to enhance work performance at the job site [1]. The goals of work rehabilitation programs are to (1) promote a safe and timely return to work, (2) help prevent future injury and rehabilitate the current injury, (3) increase the worker’s self-confidence through the resumption of their role as a worker, (4) and improve quality of life [1].

In work rehabilitation, multidisciplinary and interdisciplinary programs utilize different team approaches involving multiple disciplines to varying degrees, with interdisciplinary programs having more involvement and communication among team members. More specifically, multidisciplinary work rehabilitation interventions focus on the physical, social, psychological, and work-related aspects of care offered by a variety of clinicians from different backgrounds [2]. Multidisciplinary programs include clinicians working in parallel with one another and goal development is completed independently by each clinician, with little or no overlap between disciplines [3]. Within an interdisciplinary program, clinicians collaborate directly within the context of the program, with common goals and an integrated approach to care (e.g., team meetings and ongoing team dialogue) [3]. These interventions target psychological (e.g., fear and anxiety) and social factors (e.g., workplace social support) in addition to the physical aspects of an injury as these factors can contribute to the injury and may affect disability [2, 4].

Prior studies provide support for utilizing a multidisciplinary approach to facilitate RTW [5–8] and have looked at many factors and outcomes relating to RTW [5–10], such as intervention characteristics [5–8], prognostic factors [7, 9, 10], and disability level [6, 7, 10]. However, there is little or no prior research examining other important indicators, such as interdisciplinary intervention, the level of a patient’s RTW status (e.g., not working, new job, or ongoing restrictions) and lifting capacity/strength [see exception, 11]. For example, two systematic reviews [12, 13] reported that the level of a patient’s RTW status (e.g., not working, a new job, or ongoing restrictions) at the time of discharge following a work rehabilitation program was not identified and called for studies to do so.

Moreover, physical strength, defined in the current study as lifting capacity in relation to job tasks, is an important aspect in patient health [14–16] and may influence a patient’s RTW status [11]. Previous studies have defined strength as muscular or isometric strength [14, 16], which is different from this study as we defined strength in a functional way to assess performance of physical job demands. Functional assessments of strength are compatible with contemporary work rehabilitation models for RTW [15], but there is limited evidence to support utilizing lifting capacity as a functional measure of strength [11] and further investigation is needed. Performance of physical job demands (e.g., lifting, standing, and crouching) corresponds with aspects of RTW, as many job descriptions are based upon ability to complete lifting to varying heights and positions, such as lifting from floor to waist, waist to chest, or chest to overhead. If patients are unable to return to work without restrictions, they could potentially be terminated from their position (depending on local workers’ compensation law), which may negatively affect their overall income and quality of life as financial strain appears to create a barrier to RTW [17]. More time away from work can increase overall costs associated with the patient’s medical care, and in conjunction, increase the total costs associated with workers’ compensation (medical costs, lost productivity, and disability benefits) [18]. Surprisingly, little is known about these functional outcomes (i.e., the level of a patient’s RTW status and strength) of work rehabilitation programs and, due to the aforementioned financial and quality of life implications, the current study examined RTW status and improvements in physical ability to meet specific job demands (i.e., strength changes) following participation in a work rehabilitation program.

At this time, research has been inconclusive in determining how certain aspects of a work rehabilitation program impact RTW status [19]. For example, Fleten and Johnsen demonstrated a decrease in days off work through work rehabilitation intervention, but the reduction was not statistically significant [19]. However, in a systematic literature review, early intervention and multidisciplinary efforts appeared to be effective in treating individuals returning to work [5]. While studies have started to examine relationships between rehabilitation service utilization, timing of intervention, and duration of disability [20, 21], there is still limited evidence available to support early intervention for workplace injuries [22]. A cost-effectiveness study indicated that early intervention was related to lower medical costs, disability benefits, and productivity losses [18], which is why it is important to improve our understanding of when best to intervene for maximum gain [22]. For this reason, the current study further attempted to examine whether days off work prior to starting a work rehabilitation program was related to strength changes and RTW status.

Lastly, a recent study in Korea has indicated that the type of injury (e.g., upper extremity, lower extremity, and spine) could potentially influence a worker’s ability to RTW, with spinal injuries being the most detrimental to returning to work [9]. Granted, the generalizability of these results to Western countries may be limited due to differences in the legal and social environments, but another study conducted in Minnesota, USA corroborates these findings [10]. Specifically, Hankins and Reid reported the RTW percentages for upper extremity injuries to be 61.3%, lower extremity injuries 70.3%, and back injuries to be 55.5% [10]. Because RTW differences in type of injury were observed in the past, this study further attempted to examine whether type of injury was related to strength changes and RTW status. However, no specific predictions were made regarding these potential interactions as this was exploratory in nature.

To summarize, the current retrospective study investigated the RTW status and strength changes after completion of a comprehensive, interdisciplinary work rehabilitation program. This study further attempted to examine whether days off work prior to starting the program and type of injury were related to strength changes and RTW status.

The following research questions guided the study:

(1) Does a comprehensive work rehabilitation program affect a patient’s final RTW status?
(2) Does a comprehensive work rehabilitation program impact strength in work simulation lifting?
(3) Does the timing of intervention and type of injury influence the RTW status and strength of workers upon discharge?

It was predicted that completion of a comprehensive work rehabilitation program would be related to positive changes in RTW status and increased strength levels in workers upon discharge from the program. It was also hypothesized that the number of days off work prior to entering the program would influence patient outcomes –specifically, that delayed entry into the program would have deleterious effects on RTW status upon discharge and strength levels.

## 2 Methods

### 2.1 Study design and population

A system-wide retrospective analysis of a work rehabilitation program database examined the outcomes of the program for spine, upper quadrant, and lower quadrant injuries. This database includes all patients who had a work-related injury and were referred to the work rehabilitation program by a qualified medical practitioner. Prior to referral, the qualified medical practitioner had deemed that the patient was able to tolerate at least 1 hour of sustained, low-level physical activity for 3 to 5 days per week. We identified all patients who were admitted into the work rehabilitation program between 2006 and 2010, and included all available patients except for exclusion of records with erroneous database entries. All database records are de-identified and fully compliant with US patient confidentiality requirements, including the Health Insurance Portability and Accountability Act (HIPAA) of 1996. The Institutional Review Board at Aurora Health Care reviewed and determined that this study was intended to improve the quality of care at Aurora and did not constitute human subject research; therefore, this study was exempt from approval and did not require Aurora IRB oversight.

After exclusion of a small number of patients with erroneous database entries (*n* = 22), the final sample consisted of 495 participants (M_age_ = 44.44 years, SD = 10.13) of which 375 (76%) participants were male. Demographic variables collected included gender, ethnicity, and age. Clinical characteristics collected included the following: type of injury, number of days off work prior to the program, number of visits, and surgery status. For the type of injury, spine diagnoses included injuries to the cervical spine, thoracic spine, lumbar spine and sacrum; upper quadrant included injuries to the shoulder, elbow, forearm, wrist, and hand; and lower quadrant included injuries to the hip, knee, foot, and ankle. Further details of the study population’s demographic and clinical characteristics can be found in Tables 1 and 2. Patient outcome data included work status and lifting capacity while completing work simulation tasks pre- and post-work rehabilitation participation. The data was analyzed to determine if there was a relationship between the program and patient outcomes as measured by changes in strength and RTW status.

**Table 1:** Distribution of study population (N = 495)

Characteristic	Number	Percent
Gender	492
Male	375	76.22
Female	117	23.78
Ethnicity	491
African American	99	20.16
Caucasian	321	65.38
Hispanic	62	12.63
Other	9	1.83
Injury	489
Spine	155	31.70
Upper Quadrant	246	50.31
Lower Quadrant	85	17.38
Other	3	0.61
Surgery Status	448
Non-Surgical	155	34.60
Surgical	293	65.40
Work Level Upon Admission	486
Work with Restrictions	153	31.48
Work with No Restrictions	2	0.41
Not Working	331	68.11
Work Level Upon Discharge	488
New Occupation Capacity	28	5.74
Work with Restrictions	146	29.92
Work with No Restrictions	237	48.57
Not Working	77	15.78

**Table 2:** Descriptive statistics of the study population

Variable	N	*n*	Mean	Std. Deviation	Range
Age	490	–	44.44	10.13	19–68
Type of Injury	484
Spine Injuries	–	155	41.96	10.48	21–68
Upper Quadrant Injuries	–	244	46.25	9.84	20–66
Lower Quadrant Injuries	–	85	44.02	9.09	19–63
Number of Visits	481	–	23.48	11.62	1–72
Type of Injury	475
Spine Injuries	–	152	23.89	12.55	2–65
Upper Quadrant Injuries	–	239	23.79	10.88	3–72
Lower Quadrant Injuries	–	84	22.38	12.09	1–50
Days Off Prior to Program	483	–	145.12	313.33	0–5410
Type of Injury	477
Spine Injuries	–	151	176.85	479.31	0–5410
Upper Quadrant Injuries	–	242	134.26	214.95	0–1958
Lower Quadrant Injuries	–	84	124.24	132.90	0–726
Initial Strength (lbs.)	465	–	25.12	17.60	0–95
Type of Injury	451
Spine Injuries	–	140	30.11	18.30	0–95
Upper Quadrant Injuries	–	229	19.56	13.76	0–60
Lower Quadrant Injuries	–	82	32.62	21.16	0–95
Discharge Strength (lbs.)	459	–	42.37	23.38	0–115
Type of Injury	451
Spine Injuries	–	140	47.07	24.51	0–110
Upper Quadrant Injuries	–	229	36.94	21.05	0–100
Lower Quadrant Injuries	–	82	51.29	23.58	0–115

### 2.2 The work rehabilitation program

The work rehabilitation program is comprehensive and utilizes an interdisciplinary approach focusing on work simulation, cardiovascular activity, and overall body strengthening, with an emphasis on returning the worker to their prior occupation to reduce costs, treatment time, and lost work days. The work rehabilitation program most closely resembles a work hardening program, and the interdisciplinary team typically includes physical therapy, occupational therapy, the referring physician, and case managers along with the inclusion of licensed athletic trainers, psychologists, and vocational counselors as deemed appropriate. The interdisciplinary team functions within the same rehabilitative space and interacts daily for patient evaluation and modifications to the plan of care. The patient attending the program is the focal point of the team, and coordination with employers is also included as part of the program. Typically, patients participate in the program three to five days a week, for an average of one to three hours each treatment session. The work rehabilitation program is offered at a not-for-profit healthcare system in Wisconsin, U.S.A.

### 2.3 Outcome measures

Patients’ work level (RTW status) and strength were the primary outcomes examined in this study. Work level upon admission and discharge was classified into four levels: “new occupation capacity”, “work with restrictions”, “work with no restrictions”, and “not working”. The work rehabilitation program defined “new occupation capacity” as the patient being able to meet their physical job demands, but unable to return to their specific job due to extraneous factors, such as the job being eliminated. The first three levels were combined and defined as “working” for analysis in comparison to “not working”. Patients’ strength was measured while completing work simulation tasks pre- and post-work rehabilitation program participation utilizing the Ergoscience methodology for lifting tasks [23]. Patients with upper quadrant, cervical spine, and thoracic injuries lifted a crate loaded with weight from waist to eye level, and patients with lower quadrant and lumbar spine injuries completed floor to waist lifting. Strength was measured as the number of pounds lifted during the lifting assessment.

### 2.4 Statistical analysis

Descriptive and frequency statistics were used to summarize the characteristics of the study population. McNemar’s Test was performed to compare the change in work level upon admission to work level upon discharge. Paired-samples *t*-tests were conducted to compare pre- and post-program strength scores. The Shapiro-Wilk test was used to check normality for the distribution of the number of days off work prior to the program. Due to the non-normal distribution, a Mann-Whitney *U* test was used to compare the number of days off work prior to beginning the program for the two RTW status groups (working vs. not working). One extreme outlier in the data set (days off work >5,000) was excluded prior to conducting the Mann-Whitney *U* test. Regression analysis was performed to evaluate the relationship between the type of injury (spine, upper quadrant, and lower quadrant), the number of days off work prior to beginning the rehabilitation program, and strength (measured as lifting capacity) upon discharge. Statistical significance was set at *p* < .05. The analyses were performed with SAS software, version 9.4 (SAS Institute Inc., Cary, NC, USA).

## 3 Results

As predicted, completion of a comprehensive work rehabilitation program appears to be related to improved RTW status of participants. Table 3 shows that more participants were working at the end of the program (*n* = 406, 83.9%) than at the beginning (*n* = 153, 31.6%). The RTW rates at the work rehabilitation program, broken down by type of injury, were 84.4% for upper quadrant injuries, 81.0% for lower quadrant injuries, and 84.8% for spine injuries. Furthermore, as shown in Table 4, the comparison of the status of the workers (working vs. non-working) before and after the rehabilitation program showed that a significantly higher number of participants returned to work (*p* < 0.0001).

**Table 3:** RTW status upon admission and discharge (N = 495)

	Admission	Discharge
RTW Status	*n* (%)	*n* (%)
Working	153 (31.6)	406 (83.9)
Spine Injuries	51 (33.8)	128 (84.8)
Upper Quadrant Injuries	85 (35.0)	205 (84.4)
Lower Quadrant Injuries	16 (19.0)	68 (81.0)

**Table 4:** McNemar’s test comparing the change in work level upon admission to work level upon discharge

		Upon Discharge		
		Not Working	Working	Total
Upon	Not Working	66	265	331
Admission	Working	11	144	155
	Total	77	409	*p* < 0.0001

^*^Note: The pattern of change was significant (*p* < .0001).

**Fig.1:**
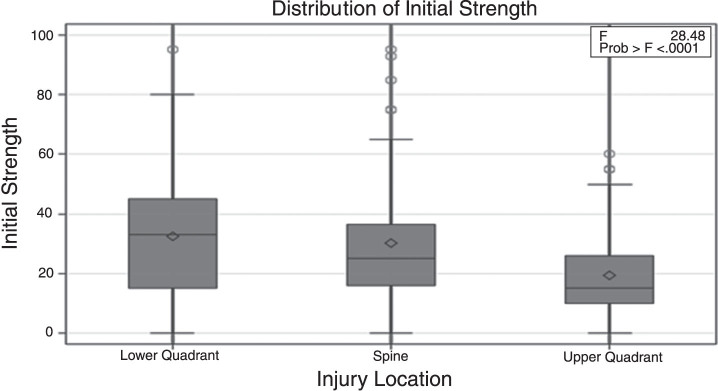
Comparison of initial strength for the three injury locations.

**Fig.2:**
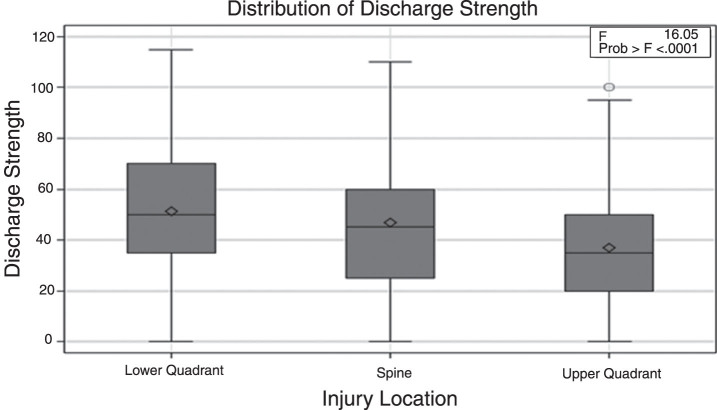
Comparison of discharge strength for the three injury locations.

As displayed in Figs. 1 and 2 and Table 5, mean strength for the three types of injuries (spine, upper quadrant, and lower quadrant) was higher at the time of discharge compared to the strength at admission (30.11±18.30 vs. 47.07±24.51, *p* < 0.0001; 19.56±13.76 vs. 36.94±21.05, *p* < 0.0001 and 32.62±21.16 vs. 51.29±23.58, *p* < 0.0001). This strength change reflects an improvement in lifting capacity while completing a work simulation task.

**Table 5:** Changes in strength scores upon admission and discharge of work specialty rehabilitation program

Injury	Admission Strength^†^	Discharge Strength^†^	Mean Difference^†^	*p*-value
	*M* (*SD*)	*M* (*SD*)	*M* (*SD*)
Spine	30.11 (18.30)	47.07 (24.51)	16.96 (19.19)	<0.0001
Upper Quadrant	19.56 (13.76)	36.94 (21.05)	17.39 (15.23)	<0.0001
Lower Quadrant	32.62 (21.16)	51.29 (23.58)	18.67 (1.91)	<0.0001

^†^Measured as number of pounds (lbs.) lifted during work simulation task.

When examining the number of days off work prior to beginning the program, mean rank for the “not working” group (282.80) was higher than the mean rank for the “working” group (229.47), indicating that the participants who did not return to work had had more days off work prior to beginning the program. The difference between these two groups in the number of days off work prior to beginning the program was found to be statistically significant (*U* = 11757, z = –3.152, *p* = 0.002).

Multivariate analysis (Table 6) for strength at the time of discharge showed that none of the variables (type of injury and days off work) were associated with strength outcomes.

**Table 6:** Regression analysis for strength upon discharge

Variable	Standardized Coefficients	*t*-value	*p*-value
Constant	43.130	11.04	<0.001
Type of Injury^†^	0.326	0.16	0.870
Days Off Prior to Program	–0.012	–0.71	0.476
Type of Injury^†^ x Days Off Prior to Program	0.001	0.14	0.885

Dependent Variable = Strength upon Discharge; Model Summary: *R*^2^ = 0.008, *F* = 1.14, *p* = 0.33, ^†^1 = Spine, 2 = Upper Quadrant, 3 = Lower Quadrant.

## 4 Discussion

Supporting the hypotheses, results showed that completion of a comprehensive, interdisciplinary work rehabilitation program was associated with improved RTW status of the worker upon discharge from the program, with more patients having returned to work. The RTW rate following participation in the work rehabilitation program was higher than what has been previously reported in a retrospective database study in Minnesota (83.9% vs. 62.3%) [10]. The RTW rates were consistently high across all three types of injuries (spine, upper quadrant, and lower quadrant), which is contrary to what has been previously reported [9, 10]. Specifically, the RTW rates at the work rehabilitation program, broken down by type of injury, were 84.4% for upper quadrant injuries, 81.0% for lower quadrant injuries, and 84.8% for spine injuries, all of which are higher than what has been previously reported for the injury types (61.3%, 70.3%, and 55.5%, respectively) [10]. Future research is needed to replicate the current findings and examine causal relationships with more robust studies.

Supporting the hypotheses, results revealed that completion of a comprehensive work rehabilitation program was related to significantly increased strength levels upon discharge from the program. This strength change reflects an improvement in lifting capacity while completing a work simulation task. Notably, the increase in strength was evident among all three types of injuries (spine, upper quadrant, and lower quadrant). This finding expands upon prior research that has examined many factors and outcomes relating to RTW but has not accounted for other functional outcomes of work rehabilitation programs (e.g., strength) [9, 10]. Although strength has been found to be an important characteristic in patient health [14–16] and may influence a patient’s RTW status [11], limited research exists within the context of work rehabilitation programs to corroborate this outcome and further research examining functional outcomes of work rehabilitation programs may be fruitful.

Supporting the hypotheses, results illustrated that more days off work prior to starting the work rehabilitation program may have deleterious effects on patients’ RTW status upon discharge. This is consistent with findings in a previous systematic literature review in which early intervention appeared to be effective in treating workers [5], and suggests that early intervention is a vital component in treating workers for a successful return to work.

Contrary to the hypotheses, it was found that strength levels upon discharge were not associated with the number of days off work prior to entering the program. The results showed that the type of injury was not related to strength at the time of discharge, either. In this study, delayed entry into the rehabilitation program does not appear to have deleterious effects on strength upon discharge as participants experienced improvements in strength regardless of the duration off work. As previously noted, limited research exists within the context of work rehabilitation programs to support this outcome and further research examining the ideal timing of intervention may be beneficial.

It is important to note that there were limitations of the current study that need to be addressed in future research. First, there were minimal inclusion and exclusion criteria utilized for this study; individuals were admitted into the work rehabilitation program based solely upon provider referral and having a work injury. Due to the lack of standardized inclusion and exclusion criteria, some patients may have been inappropriate referrals and the referral nature of the program may be a potential source of bias due to provider treatment preferences. For example, a surgeon may refer a patient to the work rehabilitation program to avoid surgery; however, surgery may be the appropriate intervention. Another limitation of the study exists within the ethical confines of the medical treatment. Due to ethical concerns and the retrospective design, potential participants were not placed into a control group to determine if these individuals would increase their strength and RTW status without an intervention. As a result, group comparisons (rehab versus no rehab) cannot be made to examine differences in outcomes between the worker who completed the program and those who never received rehabilitation services. Due to the lack of a control group, lack of randomization, and inability to control for confounding factors, causal relationships cannot be determined in this study and the results should be viewed as tentative and interpreted cautiously. Lastly, the study was also limited by a lack of discharge criteria. No standardized criteria were utilized to determine discharge, such as determining a plateau and consistent job task testing across the variety of sites, which may affect both the number of visits for each diagnosis, as well as appropriate discharge date. Thus, it is possible that some patients may have remained in the work rehabilitation program longer than necessary.

Based on the results of the study, further research is indicated. Subsequent more robust studies (e.g., quasi-experimental and experimental studies), including an evaluation of the intervention’s working mechanism, are warranted and will be helpful to determine how outcomes for workers can be improved. A recent systematic review supports extending follow-up to one year post-RTW [12] and an exploratory study found that it is valuable to look beyond RTW as a single event [24]; therefore, longitudinal research evaluating RTW outcomes at varying intervals would be beneficial to determine long-term RTW status of the individuals who participated in the program. In addition, research evaluating time since surgery in relation to the initiation of the work rehabilitation program may be beneficial in determining appropriate intervention timeframes. Lastly, it might be of value to examine potential predictive factors (e.g., patient characteristics, type of injury, time off work prior to the program, work-related characteristics, etc.) of RTW. This was not examined in this study and may be a topic for future research.

### 4.1 Conclusion

Overall, these findings suggest that the work rehabilitation program can potentially be effective and may produce the intended results for the patients who completed the program by helping the patient RTW and significantly increasing their strength as well. Also, results illustrated that delayed entry into the work rehabilitation program may have deleterious effects on patients’ RTW status at discharge. Early intervention appears to be a critical component in treating patients with a work-related injury. Lastly, it was found that strength levels upon discharge were not associated with the type of injury or the number of days off work prior to entering the program.

## Conflict of interest

None to report.
